# Dr. Budge on the Extirpation of the Salivary Glands in Animals

**Published:** 1844-01-27

**Authors:** 


### DR. BUDGE ON THE EXTIRPATION OF THE SALIVARY GLANDS IN ANIMALS.

In his researches on the saliva, M. Budge constantly found that fluid alkaline, before as well as after a repast. This alkaline reaction was found to exist in animals even after a prolonged fast. To ascertain what effect the salivary glands had on the alkalinity of the buccal secretions, M. Budge extirpated the whole of the salivary glands of a dog, viz. the parotids, submaxillary, and sublingual glands of both sides. The dog survived the operation, but the buccal secretions were constantly found alkaline. When the dog was killed at the end of a month, the fluid of the stomach was found slightly acid. The same operation was repeated in a rabbit, and with the same success,—the buccal secretions remained alkaline.—*Ibid, from Med. Zeitung.*

